# Coupled InVEST–MGWR modeling to analyze the impacts of changing landscape patterns on habitat quality in the Fen River basin

**DOI:** 10.1038/s41598-024-64012-9

**Published:** 2024-06-07

**Authors:** Juemei Wu, Yanjun Hou, Zheng Cui

**Affiliations:** 1 School of Geography Science, Liaoning Normal University, Dalian, 116029 China; 2 Department of Geography, Xinzhou Teachers University, Xinzhou, 034000 China; 3 School of Geographical Sciences, Shanxi Normal University, Taiyuan, 030000 Shanxi China; 4 School of Management, Liaoning University of International Business and Economics, Dalian, 116052 Liaoning China

**Keywords:** Fen River Basin, Landscape index, Habitat quality, MGWR model, Ecosystem services, Environmental impact

## Abstract

The present study employed remote sensing images of the Fen River Basin from 2005, 2010, 2015, and 2020 as the primary data source. The software ENVI, ArcGIS, and Fragstats 4.2 were utilized to measure the landscape pattern index of the Fen River Basin. A collinearity test was conducted to remove any redundant landscape pattern indices. Based on the selected landscape indices, the landscape pattern index values were ascertained as follows. Using the shifting window method, the landscape pattern index of the Fen River Basin was obtained. Second, the habitat quality in the Fen River Basin was assessed using the InVEST model, and the spatial autocorrelation approach was employed to confirm that the habitat quality was spatially autocorrelated. Finally, the spatial impacts of landscape pattern indices on habitat quality were examined using the MGWR model. The results show that (1) the Fen River Basin's overall habitat quality declined between 2005 and 2020; however, the deterioration slowed with time and had a typical "poor in the middle and high around the margins" spatial distribution. The habitat quality of the low-value area continued to increase, the habitat quality of the lower-value area decreased annually, the habitat quality of the middle-value area decreased and then increased, the habitat quality of the higher-quality area tended to increase, decrease, and then increase again, and the habitat quality of the high-quality area decreased annually. (2) The fit of the MGWR model was greater than those of the OLS and traditional GWR models, and it was able to more clearly illustrate the various roles that landscape pattern indices and habitat quality play in one another. (3) Changes in landscape patterns had a major impact on habitat quality; habitat quality was positively impacted by PD and AI, negatively impacted by MESH, and had positive and negative bidirectional effects from CONTAG and AI.

## Introduction

Global urbanization has accelerated recently, as has the impact of human activity on the environment^1,2^. Numerous studies have demonstrated that both man-made and natural causes, such as changes in land use, the development of infrastructure, and agricultural output, have drastically altered landscape patterns^3–5^. In addition to having an adverse effect on biodiversity and ecosystem stability, landscape fragmentation caused by abrupt changes in landscape patterns also has a number of detrimental effects on human society, including decreased soil quality, water scarcity, and an increased frequency of natural disasters^6^. A healthy, functional ecosystem maximizes its potential for production, vitality, and ecology. It also improves the system's ecological and economic benefits, the local ecological environment, and the high-quality development of local economies^7,8^.

Changes in landscape type will alter the configuration and composition of regional landscape elements, which will disrupt the ecosystem and ultimately alter the quality of the regional ecological environment. Landscape patterns, on the other hand, include the spatial composition, distribution, configuration, and composition of the units in the landscape, among other attributes, which further affect the ecological processes of the ecosystem^9–11^. Large-scale conversions of agricultural land to construction sites have been linked to increased landscape homogenization and fragmentation, which may endanger species' ability to survive. Additionally, fragmentation of the landscape can have detrimental ecological effects on the continuity and quality of ecosystems^12,13^. The prevalent quantitative study approach in landscape ecology is describing landscape patterns and creating connections between habitat quality and landscape patterns by choosing suitable landscape indices^6,14^.

Many academics have been delving more deeply into the significance and underlying mechanisms of changes in landscape patterns on habitat quality in recent years^15–17^. Previous studies have mainly used gray correlation models, least squares (OLS), locally weighted regression (LWR) and geographically weighted regression (GWR) to explore the effects of landscape pattern index changes on habitat quality; for example, Lu Yu et al. employed a combination of geographically weighted regression (GWR) and the least squares (OLS) model to investigate the impact of habitat quality on landscape patterns in Wanning city^18–20^. Traditional linear regression techniques are still often employed in many different domains for quantitative studies^21^. The classic linear regression method, referred to as ordinary least squares (OLS), is primarily used to investigate the relationships between explanatory variables and independent variables. However, the basis for this method is the idea that statistics are most stable across the entire dataset. Additionally, when applying OLS to spatial data, it ignores nonstationarity, which could result in erroneous conclusions. This could cause erroneous conclusions to arise^16,22,23^. Geographically weighted regression (GWR), on the other hand, can reveal local properties and spatial nonstationarity of spatial data. When spatial nonstationarity is present, GWR typically yields more realistic regression equations and more accurate model predictions than OLS. However, in terms of bandwidth results, GWR finds the same optimal bandwidths for each explanatory variable; however, the roles of different explanatory variables scale differently, so different optimal bandwidths need to be carefully examined for each variable^24,25^. These problems are addressed by the multiscale geographically weighted regression (MGWR) model, which is an extension of GWR^26^. The MGWR determines the ideal bandwidth for every explanatory variable, enhancing the model's performance and illuminating the extent to which the explanatory variables are important^27,28^. The model is widely used in disciplines such as geo-economics, epidemiology, and environmental research. It expands the GWR model spatially and offers fresh perspectives on the regression results^29^.

Research on the mechanisms behind the spatial effects of landscape patterning on habitat quality has received less attention. Although the establishment of ecological corridors and improved connectivity between various sites might boost an area's biodiversity, the fragmentation of high-quality habitats dramatically diminishes faunal variety^30^. Fewer studies have focused on the precise processes via which habitat fragmentation influences habitat quality, but those that have used OLS to examine the connection between the two have revealed that alterations in landscape patterns may have a negative impact on habitat quality^31^. However, there is some duplication in the number mining, the representativeness of the variables chosen is insufficient to completely characterize the landscape pattern, and the inadequacy of OLS in spatial mining is insufficient to support the study's conclusions. The diversity of landscapes and the degree of aggregation have an impact on habitat quality in rapidly urbanizing cities, according to research conducted by some scholars on the mechanisms by which landscape pattern affects habitat quality in GWR. However, this study focused on the mechanisms by which urbanization affects habitat quality and did not summarize the effect of landscape fragmentation on habitat quality^32–34^. Additionally, few studies have examined the spatial and temporal effects of landscape fragmentation on habitat quality using geographically weighted multiscale regression models. The MGWR model offers a useful way to investigate this driving mechanism by examining the spatial scales at which the effects are observed. Scientists are interested in this spatial econometric model because of its capacity to investigate the spatial correlation of spatial variables. Thus, using this method to investigate the processes through which habitat quality is impacted by landscape fragmentation will yield insightful findings.

Using Landsat image data from 2005, 2010, 2015, and 2020, this paper examines the geographic effects of landscape pattern evolution on habitat quality in the Fen River Basin. The primary goals of this study were to (1) extract the landscape pattern index of the Fen River Basin using the moving window method, (2) identify the temporal and spatial variability of habitat quality, and (3) use the MGWR model to assess the relationships between changes in the landscape pattern index and habitat quality. Finally, this study aims to shed light on the effects of changes in landscape patterns caused by rapid urbanization on habitat quality. The understanding of the spatial effects of landscapes can be strengthened by this research.

## Data sources and methods

### Study area

**Figure 1:**
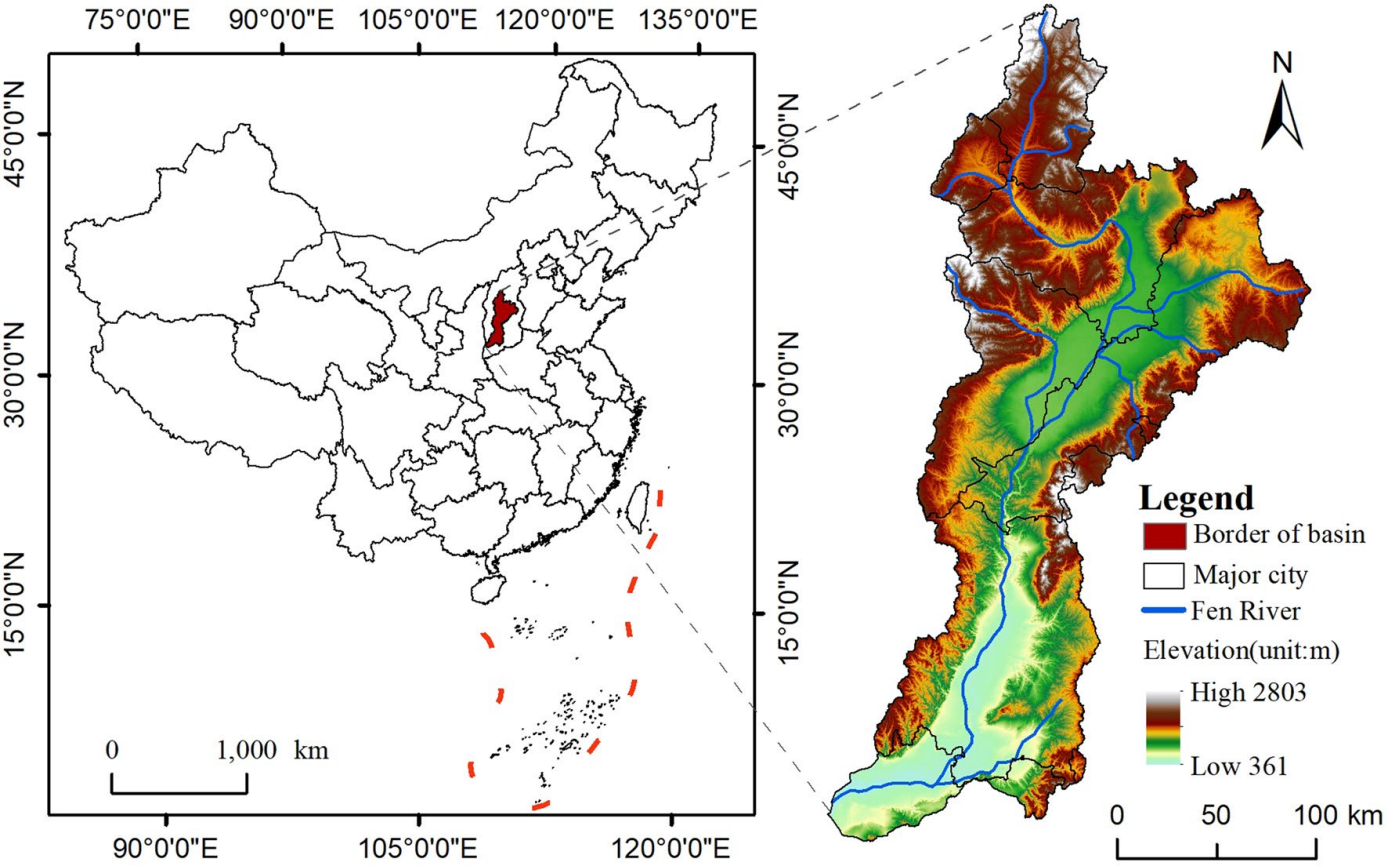
the study area. Note: The administrative boundary data of the Fen River Basin are from the Resource and Environmental Science Data Platform (https://www.resdc.cn/data.aspx?DATAID=278);the DEM data were obtained from Geospatial Data Cloud (https://www.gscloud.cn/); the map data of China are based on the GS (2022) 1873 standard map downloaded from the National Administrative Division Information Query Platform (http://xzqh.mca.gov.cn/map). ArcGIS10.7 generated the aforementioned data; the base map boundary remains unchanged.

The second-largest tributary of the Yellow River Basin, the Fen River Basin, is a significant portion of the Loess Plateau and is a part of the Yellow River Basin. The southern–central region of Shanxi Province (35°13′4 ~39°4′4″N, 110°26′42″~113°26′56″E) has a watershed area of approximately 3.97×104 km^2^, accounting for 25.3% of the province's total area (Fig. 1). The main stream runs for a total of 716 km in length, with Xinzhou, Lvliang, Yangquan, Taiyuan, Jinzhong, Changzhi, Linfen, Jincheng, and Yuncheng included in its watershed area. The main stream flows through the middle and southern parts of the province from north to south, passing through Xinzhou, Taiyuan, Lvliang, and six other cities. The tributary systems originate from the two major mountain ranges, and the terrain is complex and undulating. The Fen River basin is surrounded by the Taihang Mountains in the east and the Lvliang Mountains in the west. The topography is complicated and undulating. In general, Shanxi Province's topography is long in the north and south, thin in the east and west, and sporadically distributed in the central region. The Fen River Basin has a moderate continental monsoon climate, with an average annual temperature of 7 °C to 13.7 °C and 400–600 mm of precipitation, most of which falls in the summer and is dispersed irregularly throughout the year. Brown, meadow, and loess soils are the predominant soil types in the basin, while deciduous broad-leaved woods and coniferous forests make up the majority of the vegetation.

### Data sources and preprocessing

#### Remote sensing image data

The base data source consisted of 20-scene remote sensing images scanned by Landsat 5 TM/Landsat 8 0LI satellite sensors in 2005, 2010, 2015, and 2020, with a spatial resolution of 30 m × 30 m and less than 1% cloud cover. The spatial reference coordinate system of the images was standardized as WGS_1984_UTM_Zone_49N. The primary sources of images were the Geospatial Data Cloud (http://www.gscloud.cn/). The chosen image imaging time was between May and October to ensure the accuracy of the evaluation index. If the amount of cloud cover is insufficient to meet the study's requirements, another time period may be chosen, but in this case, the image data will be as close to the vegetation's growing season as possible. The rich spectral information of the image data facilitates the interpretation of remote sensing images (Table 1).

**Table 1:** Remote Sensing Image Data.

Number	Data	Year	Track number/Sequence number	Resolution/m
1	Landsat 5 TM	2005	125/34, 125/35, 126/33, 126/34, 126/35	30
2	Landsat 5 TM	2010	125/34, 125/35, 126/33, 126/34, 126/35	30
3	Landsat 8 0LI	2015	125/34, 125/35, 126/33, 126/34, 126/35	30
4	Landsat 8 0LI	2020	125/34, 125/35, 126/33, 126/34, 126/35	30

**Figure 2:**
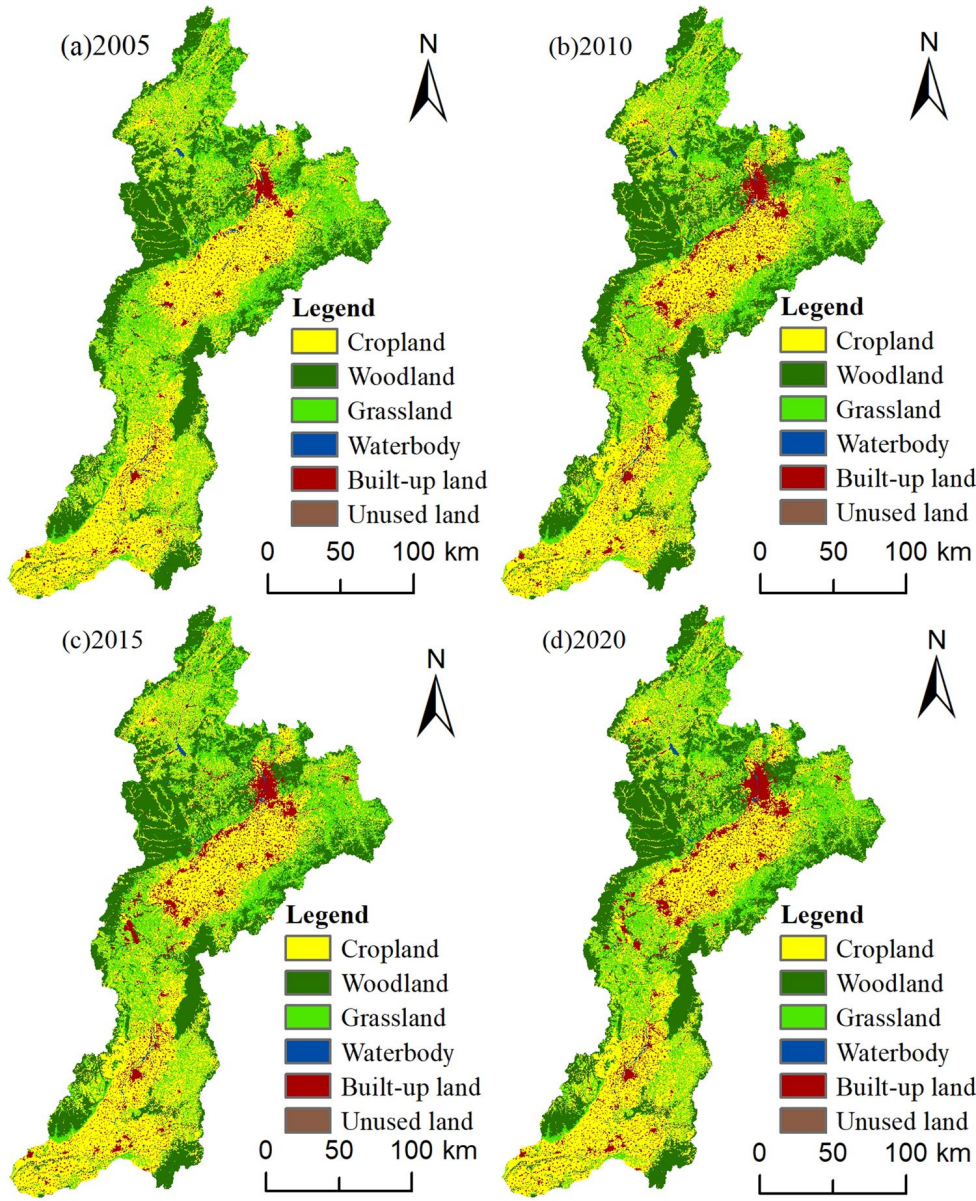
Spatial distribution of intangible cultural heritage resources in China. Note: The remotely sensed image data (http://www.gscloud.cn/) used to create this map was obtained with ArcGIS10.7 and ENVI5.6. Remote sensing photos were utilized to pre-process the map. The features from the photos were then classified using the supervised classification module in ENVI5.6 software. Finally, ArcGIS10.7 was used to create the map.

It is frequently necessary to preprocess remotely sensed images using ENVI software according to the characteristics of the remotely sensed images, which mainly include radiometric calibration, atmospheric correction, image mosaicing, and image cropping, before the supervised classification of remotely sensed images. This is done to eliminate the problems of image data overlap and radiometric brightness distortion caused by the solar altitude, topography, atmosphere, and the sensor's own photoelectric system. The Fen River Basin's actual land-use situation, in conjunction with the Third National Land Survey's classification system and the Classification of Land Use Status GB/21010-2017, divides the land into six categories: Cropland, Woodland, Grassland, Water body, Built-up land, and Unused land. The deciphering markers for these six categories are established visually based on the shape, texture, and hue of the corresponding images and are subsequently overseen by the maximum likelihood method. Then, by integrating the classifier and visual interpretation to extract the land-use information of the four phases, the maximum likelihood technique was utilized to oversee the interpretation of the remote sensing images (Fig. 2). The overall accuracy of the picture classification findings (Table 2) was more than 80%, and the kappa coefficient was greater than 0.7, indicating that the results of remote sensing image interpretation may satisfy the requirements of future research.

**Table 2:** Accuracy verification table.

	2005	2010	2015	2020
Overall Accuracy	80.45%	85.36%	98.31%	95.65%
Kappa Coefficient	0.79	0.81	0.97	0.93

#### Nonremote sensing image data

The administrative vector boundary data for the Fen River Basin were acquired from the Resource and Environmental Science Data Platform (https://www.resdc.cn) and was used for the study region. The Geospatial Data Cloud (http://www.gscloud.cn/) provided the DEM data.

### Research framework

**Figure 3:**
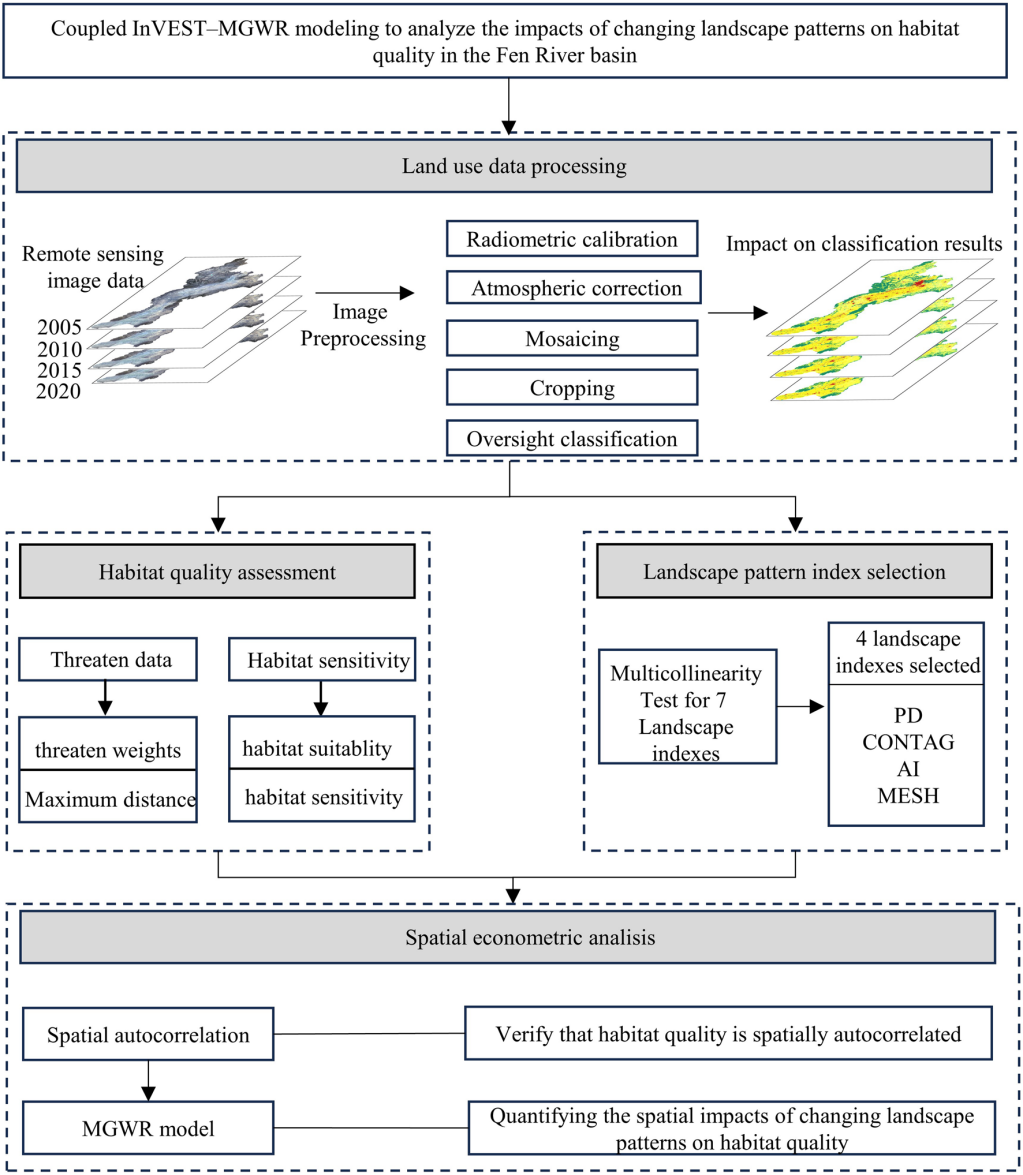
Research framework diagram.

The four main steps of this study were as follows: (1) preprocessing remote sensing images from 2005 to 2020 and obtaining image feature classification result maps using supervised classification; (2) calculating the Fen River Basin's landscape pattern index using the moving window method (MWM); (3) characterizing the temporal and spatial variations in habitat quality using the InVEST model; and (4) analyzing the landscape pattern evolution based on the MGWR model on the spatial quality of habitat. Figure 3 illustrates the study's flow chart.

### Research methods

#### Moving window method

The fundamental principle behind the moving window method is to start calculating at the first data point located in the upper left corner of the study region. The landscape index value of the complete moving window will be calculated for each window, and as the window slides, the research region will be gradually covered^35,36^.

Seven landscape pattern indices—the patch density (PD), contraction index (CONTAG), aggregation index (AI), effective mesh size (MESH), landscape division index (DIVISION), Shannon diversity index (SHDI), and split index—were chosen for this paper based on prior research and the actual conditions of the Fen River Basin (SPLIT). These indices frequently employ three perspectives—landscape fragmentation, heterogeneity, and connectivity—to characterize shifts in landscape patterns.

The next investigation avoided data repetition because the meanings of the landscape pattern indices were comparable. Before the test was run, the landscape pattern indices were normalized using ArcGIS software's fuzzy affiliation tool to remove the magnitude effect. A multicollinearity test was then run on the seven landscape pattern indices that had been chosen, with the goal of excluding landscape pattern indices that had a variance inflation factor (VIF) larger than 10. The findings are displayed in Table 3, where it can be seen that the final landscape pattern indices chosen were PD, CONTAG, AI, and MESH. The variance inflation factors (VIFs) of DIVISION, SHDI, and SPLIT were all greater than 10.

**Table 3:** Landscape pattern index covariance test results.

B	Unstandardized coefficients		Standardized coefficients	t	Significance	R^2^	F	Covariance statistics	
	Standard errors	Beta						Tolerances	VIF
(Constant)	0.730	0.032		22.694	0.000	0.029	147.403		
PD	0.199	0.037	0.068	5.421	0.000			0.177	5.638
CONTAG	0.031	0.008	0.027	3.740	0.000			0.558	1.792
AI	− 0.195	0.032	− 0.079	− 6.124	0.000			0.166	6.010
MESH	0.116	0.009	0.080	13.163	0.000			0.762	1.311
DIVISION	− 0.004	0.037	− 0.004	− 0.113	0.910			0.027	36.799
SHDI	0.113	0.041	0.069	2.794	0.005			0.046	21.676
SPLIT	− 0.092	0.061	− 0.027	− 1.516	0.130			0.088	11.398

#### InVEST model

The habitat quality module of the InVEST model was used in this study to evaluate the habitat quality of the Fen River Basin. The fundamental function of the model is derived from data on land use. The degree to which threat factors influence the quality of habitats is determined by the interaction between these factors and habitats. The degree of habitat degradation and biodiversity in the study area is reflected in the spatially quantitative assessment of the results^37^. The quality of the habitat is lower in places with high levels of anthropogenic disturbance. The following formula can be used to determine the degradation of habitat quality:

1 $$\begin{array}{c}{D}_{xj}=\sum_{r=1}^{R}\sum_{y=1}^{{Y}_{r}}\left((\frac{{\omega }_{r}}{\sum_{r=1}^{R}{\omega }_{r}}\right){r}_{y}{i}_{rxy}{\eta }_{x}{S}_{jr}\end{array}$$

where ${D}_{xj}$ is the habitat quality stress intensity index of grid *x* in land-use type* j*, *R* represents the threat factor, *R* represents the number of threat factors, and ${\omega }_{r}$ is the weight of the threat factor. The range is between 0 and 1. The closer the weight is to 1, the greater the influence on the habitat quality; ${S}_{jr}$ refers to the sensitivity of land-use type* j* to threat factor *r*. The range is between 0 and 1. The greater the value is, the stronger the sensitivity. ${i}_{rxy}$ represents the threat source value ${r}_{y}$ of grid *y*. The threat level *y* to grid *x*. The model also proposes two ways to calculate ${i}_{rxy}$, with the following formula:

2 $$\begin{array}{c}{i}_{rxy}=\left\{\begin{array}{c}1-\left(\frac{{d}_{xy}}{{d}_{r max}}\right)\left(\text{Linear decay}\right)\\ exp\left[-\left(\frac{2.99}{{d}_{r max}}\right){d}_{xy}\right]\left(\text{Exponential decline}\right)\end{array}\right.\end{array}$$

where ${d}_{xy}$ is the distance between grid* x* and grid *y* and ${d}_{r max}$ refers to the maximum influence range of the threat factors. The higher the ${D}_{xj}$ value is, the greater the impact of threat factors on habitat quality, and the greater the degree of habitat degradation.

The habitat quality assessment formula is as follows:

3 $$\begin{array}{c}{Q}_{xj}={H}_{j}\left(1-\frac{{D}_{xj}^{z}}{{D}_{xj}^{z}+{K}^{z}}\right)\end{array}$$

where ${Q}_{xj}$ is the habitat quality index of grid *x* in land-use type* j*; ${H}_{j}$ is the habitat suitability of land-use type* j*, the value range is between 0 and 1, and the closer to 1, the stronger the suitability is; *Z* is the normalized constant; and *K* is the semisaturation constant, generally 1/2 of the maximum value of ${D}_{xj}$.

This research identified cropland, built-up land, and unused land as risk factors that compromise the quality of habitat^38^. The model's input parameters include the threat variables' maximum impact distance, weight, decline type, and each land-use type's sensitivity to each danger component, among other factors. The InVEST model's user manual and pertinent research findings from earlier studies were consulted in selecting the aforementioned parameters, which are displayed in Tables 4 and 5.

**Table 4:** Threat factor parameter setting.

Threat Factors	Maximum impact distance/km	Weight	Type of recession
Cropland	6	0.6	Linear
Built-up land	9	0.9	Exponential
Unused land	4	0.4	Linear

**Table 5:** Sensitivity of different landscape types to habitat threat factors.

Landscape types	Habitat suitability	Sensitivity		
		Cropland	Built-up land	Unused land
Cropland	0.5	0.3	0.7	0.1
Woodland	1.0	0.8	0.6	0.2
Grassland	1.0	0.5	0.6	0.6
Water body	0.7	0.4	0.7	0.4
Built-up land	0.0	0.0	0.0	0.0
Unused land	0.0	0.0	0.0	0.0

#### Spatial autocorrelation

The spatial autocorrelation of habitat quality must be measured prior to applying spatially weighted regression. By computing the autocorrelation coefficients and evaluating their significance, spatial autocorrelation seeks to quantitatively characterize the spatial correlation and distribution of samples by ascertaining whether the value of an attribute of one sample point under study is spatially related to the value of the same attribute of other sample points in the domain^39^. The geographical clustering pattern, discrete pattern, and stochastic pattern of habitat quality in the Fen River Basin were identified in this study using global autocorrelation analysis. Moran's I index was used to measure the spatial autocorrelation of habitat quality in the Fen River Basin from 2005 to 2020 based on the spatial location and quality of the habitat. The index's value ranged from -1 to 1, where a value of 1 denotes a completely positive spatial autocorrelation, meaning that the observed values are positively correlated with the surrounding observations, and a value of -1 denotes a completely negative spatial autocorrelation, meaning that the observed values are negatively correlated with the surrounding observations. Zero indicates the absence of spatial autocorrelation. Particular equation:

4 $$\begin{array}{c}I=\frac{n\sum_{i=1}^{n}{\sum }_{j=1}^{n}{\omega }_{ij}({x}_{i}-\overline{x })({x}_{j}-\overline{x })}{\sum_{i=1}^{n}{\sum }_{j=1}^{n}{\omega }_{ij}\sum_{i=1}^{n}{({x}_{i}-\overline{x })}^{2}}\end{array}$$

where* n* is the number of elements, ${x}_{i}$ and ${x}_{j}$ are the attribute values of elements* i* and* j*, $\overline{x }$ is the average of the attribute values, and ${\omega }_{ij}$ is the spatial weight between elements *i* and* j*.

#### MGWR model

Multiscale Weighted Geographic Regression. A localized version of linear regression called multiscale geographically weighted regression (MGWR) is used to model relationships that vary spatially^40^. It is built using geographically weighted regression (GWR), a local regression model that permits spatial variation in the explanatory variable coefficients^41^. By permitting the model to have distinct coefficients at various geographic regions, MGWR, in contrast to conventional global linear regression models, improves the model's ability to capture the spatial heterogeneity of the data. By considering the geographic location of the data, this local regression model can increase the goodness-of-fit and prediction accuracy of the model. The fundamental idea is to break the entire study area into several distinct sections, each of which is then subjected to a separate linear regression analysis. In this manner, the location of the region and the features of its surroundings may be used by the regression model for each small region to determine its coefficients. Ultimately, an overall regionally weighted regression model with multiscale properties can be formed by combining the local regression models of various tiny regions. The precise equation is as follows:

5 $$\begin{array}{c}y={\beta }_{0}\left({u}_{i},{v}_{i}\right)+\sum_{i=1}^{n}{\beta }_{bwj}\left({u}_{i},{v}_{i}\right){x}_{ij}+{\varepsilon }_{i}\end{array}$$

where ${\beta }_{0}\left({u}_{i},{v}_{i}\right)$ is the intercept constant of the regression equation, *n* is the total number of observations, ${\beta }_{0}\left({u}_{i},{v}_{i}\right)$ is the spatial location of observation *i*, ${\beta }_{bwj}$ is the regression coefficient of covariate *j*, ${x}_{ij}$ is the covariate, and ${\varepsilon }_{i}$ is the error term.

## Results and analysis

### Analysis of the spatial and temporal evolution of habitat quality

The suitability of regional ecosystems can be reflected in the habitat quality index; the higher the value is, the better the habitat quality, and vice versa. The habitat quality index was used to calculate the overall habitat quality of the Fen River Basin. The mean values for the years 2005, 2010, 2015, and 2020 were 0.6388, 0.6199, 0.6168, and 0.6143, respectively. The average value of the index was greater than 0.6, indicating that the habitat quality of the Fen River Basin was better. The habitat quality index decreased the fastest between 2005 and 2010 and then slowed between 2010 and 2020. Overall, the habitat quality of the Fen River Basin was better. The habitat quality index decreased most quickly between 2005 and 2010 and then slowed between 2010 and 2020. Overall, the habitat quality of the Fen River Basin declined annually.

Referring to the pertinent literature^19^, we employed the natural discontinuity method to classify habitat quality into five grades—low (0–0.3), low (0.3–0.5), medium (0.5–0.7), high (0.7–0.9), and high (0.9–1.0)—and counted the area and percentage of each grade to systematically analyze the overall change in habitat quality in the Fen River Basin over time. and the proportion of each habitat grade. Table 6 displays the fraction of the Fen River Basin that has varying habitat quality classifications each time. In 2005, 2010, 2015, and 2020, the habitat quality of the high-value area was the most widely distributed throughout the entire basin, spanning 20723.54 km^2^, 20360.64 km^2^, 20306.94 km^2^, and 20252.41 km^2^, respectively, and, when viewed from a time series perspective, the habitat quality of this habitat grade was the most widely distributed throughout the entire basin, spanning 20723.54 km^2^, 20360.64 km^2^, 20306.94 km^2^, and 20252.41 km^2^, respectively. According to the time series, the percentage of this habitat quality level shows a decreasing trend year by year; in 2020, the area decreased by 471.13 km^2^ compared to that in 2005, a decrease of 1.21%; in contrast, the proportion of the habitat quality level in the higher-value area remained unchanged, and the area generally exhibited an "increase–decrease–increase" trend; in 2020, the area of this value area increased by 1.52 km^2^ compared to that in 2005. The habitat quality of the medium-value zone generally declined, and the area of the zone at the end of the study decreased by 0.15% compared to that at the beginning of the study. The habitat quality of the lower-value zone generally declined, and in 2020, the percentage was 39.66%, with an area of 15,546.40 km^2^, and the area of this value zone in 2020 decreased by 2.05% compared with that of the area in 2005. The habitat quality of the lower-value zone improved annually, and the final area of the study area increased by 3.4% compared to its initial value.

**Table 6:** Area and percentage of various habitat levels in the Fen River Basin from 2005 to 2020.

Habitat quality level	2005		2010		2015		2020	
	Area/km^2^	Percentage/%	Area/km^2^	Percentage/%	Area/km^2^	Percentage/%	Area/km^2^	Percentage/%
Low	1728.32	4.41	2750.62	7.02	2924.79	7.46	3062.48	7.81
Relatively low	16,350.51	41.71	15,752.33	40.18	15,632.74	39.88	15,546.40	39.66
Medium	328.21	0.84	266.16	0.68	265.69	0.68	268.89	0.69
Relatively high	69.52	0.18	71.48	0.18	69.97	0.18	71.04	0.18
High	20,723.54	52.87	20,360.64	51.94	20,306.94	51.80	20,252.41	51.66

**Figure 4:**
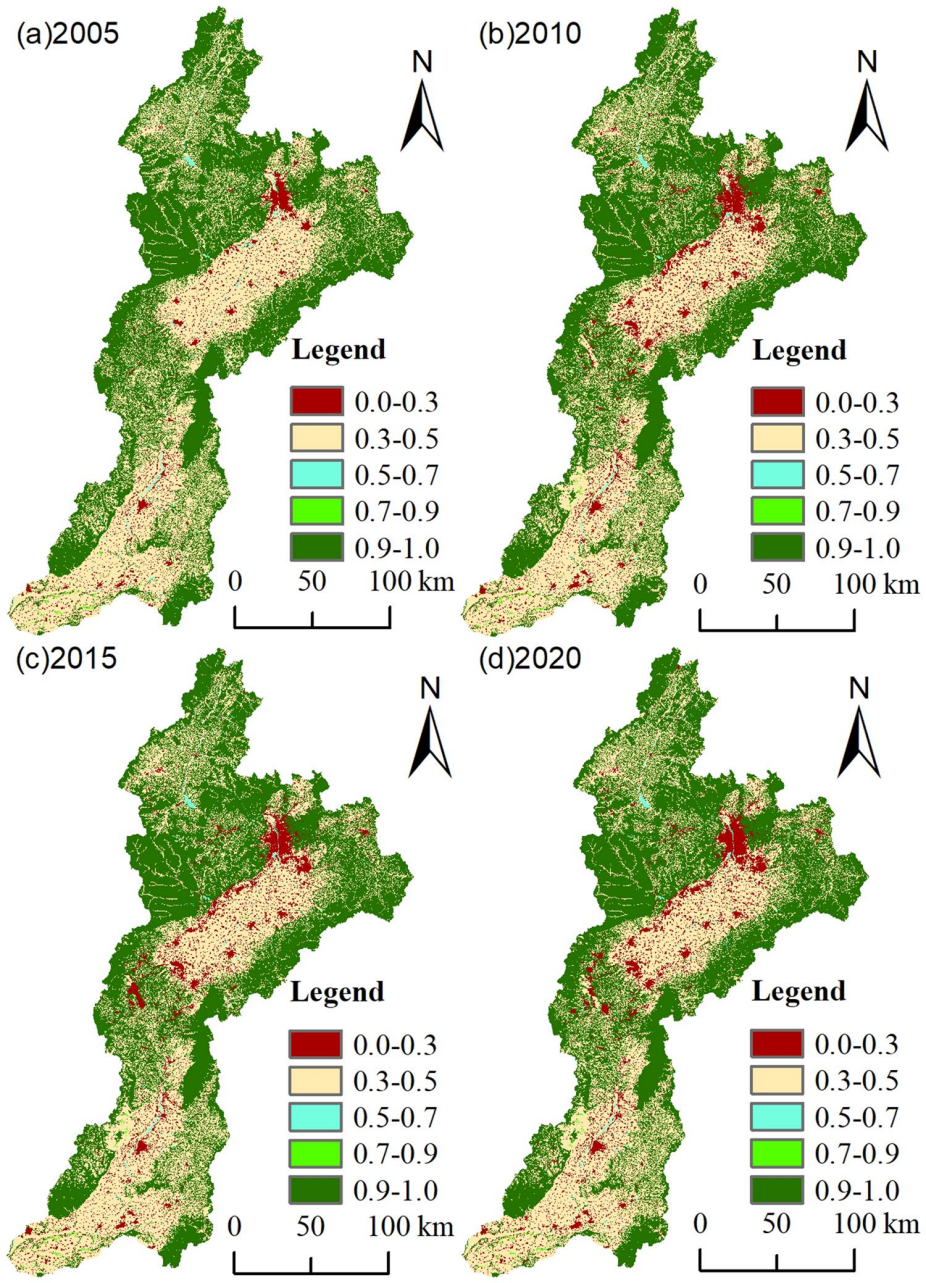
Spatial and temporal distribution of habitat quality, 2005–2020. Note: This map was created using the Habitat Quality module in InVEST 3.10.2 and ArcGIS 10.7, and it is based on the outcomes of feature classification of remote sensing imagery.

The spatial distribution pattern of habitat quality in the Fen River Basin from 2005 to 2020 was similar, and there was no significant abrupt change in this pattern (Fig. 4). This suggests that the overall trend of habitat quality in the Fen River Basin over the previous 15 years was relatively stable, with a "low in the middle and high around the edges" in the overall trend and regional variability in the spatial distribution. Over the last 15 years, the geographical distribution of habitat quality has remained largely steady, exhibiting an overall tendency of "high at the periphery and low in the middle", with some noticeable regional variations in the distribution.

The habitat quality of high-value areas, such as Xiangning County in the country's southern mountains, Jiaokou city in the country's center, Wenshui County, Jiaocheng County, Ningwu County, and other edges, is primarily concentrated at the edges of the study area with high vegetation cover. These areas have a high degree of overlap with the high-value area of the DEM, with higher terrain, less interference from human activity, and with the primary land-use types of woodlands and grasslands. The higher value zones are more widely distributed geographically, have a lower proportion of habitat quality, and are primarily composed of grasslands. The primary land-use types are dominated by water in the medium-value zone, which is dispersed throughout the watershed. Over 40% of the total watershed area is classified as a poor-value zone, with the majority of it located in the south-central core area of agricultural production. This area is characterized by flat terrain, high levels of human activity, and a predominance of arable land as the primary land-use type. A smaller portion of the poor-value zone is also found in the upper part of the watershed, which is unevenly distributed across Ningwu County, Jingle County, and Shouyang County. As urbanization progresses, the areas with the lowest habitat quality are primarily located in the Fen River Basin, which serves as the center of economic development. This area is characterized by a high intensity of disturbance from human activity, a propensity for outward expansion, and a predominant land-use type of construction land.

### Spatial autocorrelation test for habitat quality

Prior to executing the MGWR model, more research on the spatial autocorrelation of habitat quality in the Fen River basin is needed. Specifically, the study area was first divided into a 3000 m × 3000 m grid using ArcGIS, and the habitat quality was extracted for each grid using the Multi-Value Extraction to Points tool. After that, the descriptive statistical parameters of the spatial effect of habitat quality were further measured using GeoDa software (Table 7). The Moran’s I values from 2005 to 2020 were all greater than 0.7, the Z values were all greater than 25, and the P values were all less than 0.01; these results suggest that the habitat quality in the Fen River Basin has a high spatial autocorrelation.

**Table 7:** Descriptive spatial autocorrelation parameters of habitat quality in the Fen River Basin from 2005 to 2020.

Year	Moran, s *I*	Z value	P
2005	0.769	28.88	0.001
2010	0.774	28.9	0.001
2015	0.773	28.97	0.001
2020	0.771	28.97	0.001

### Comparative analysis of the MGWR, GWR and OLS models

A multiscale geographically weighted regression (MGWR) model was used to reveal the influence of landscape pattern index changes on the spatial distribution of habitat quality. To thoroughly investigate the impact of landscape pattern changes on habitat quality in the Fen River Basin, a 3000 m×3000 m fishing grid was created using ArcGIS. Landscape pattern indices and habitat quality were extracted for each cell in the grid. Whereas the traditional geographically weighted regression (GWR) model could only reflect the mean of the scale of action of each variable, the multiscale geographically weighted regression (MGWR) model was able to reflect the differentiated scale of action situation of different variables on the habitat quality of the Fen River Basin. The modified Akaike informativeness criterion (AICc) was used as a criterion for model selection because of the complexity of the model. According to the global regression model calculation results under the MGWR model, GWR model, and ordinary least squares (OLS) regression (Table 8), the R^2^ result was primarily used to measure the model's ability to explain the variability of the data, and the closer the R^2^ was to 1, the stronger the model’s explanatory power was. Better data simulation is achieved by the model with a lower AICc score. According to the model comparison results, the MGWR model had the lowest AICc value of 4703.74, followed by the GWR model with 9421.96 and the OLS model with 10043.75. The largest R^2^ was 0.862 for the MGWR. In conclusion, in terms of fitting efficacy, the MGWR model performed better than the GWR and OLS models.

**Table 8:** Comparison of the MGWR, GWR and OLS models.

Model	AICc	R^2^	Adj. R^2^
MGWR	4703.74	0.862	0.852
GWR	9421.96	0.561	0.561
OLS	10,043.75	0.126	0.123

### Spatial impacts of changing landscape patterns on habitat quality

The MGWR model can capture differences in the spatial extent of the effects of the various explanatory variable factors and the magnitude of the effects of the various explanatory variables on habitat quality. The bandwidths of the variables in the GWR and MGWR models measure the magnitude of the effects of the explanatory variables. Additionally, the effects of various bandwidth parameters differ in terms of geographical heterogeneity.

**Figure 5:**
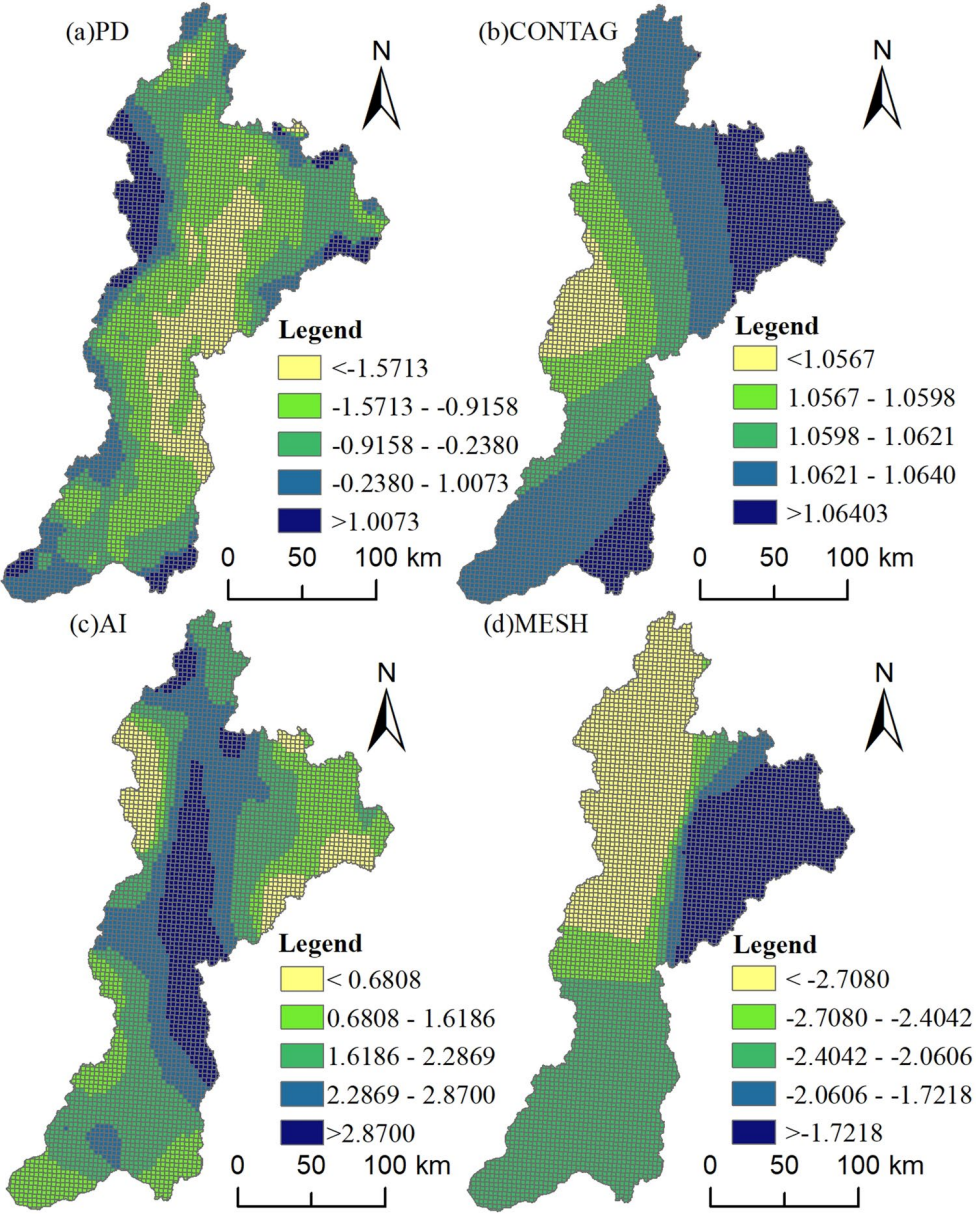
Spatial distribution of the MGWR model coefficients.

The bandwidth of PD, 44, which accounted for 11% of all samples, as shown in Figure 5 and Table 9, indicates that the scale of action was approximately 4314 km^2^, and the considerable spatial heterogeneity suggests that the quality of habitats was susceptible to changes in the density of patches. With the middle plain as the center and gradually increasing in all directions, the results demonstrated that patch density had both positive and negative effects on habitat quality in various regions. This was primarily demonstrated by the stronger negative correlation that increased with the proximity of urban construction land and the more significant positive correlation that increased with the distance of urban construction land from the urban center. Habitat quality and patch density were significantly positively correlated in areas with high habitat quality values. The strongest negative correlation is observed in the middle reaches of the region, which are distributed in the core areas of the plains' middle reaches, including Taiyuan, Qingxu, Pingyao, Jiexiu, and Lingshi. These areas are also part of the watershed's economic-development core, where the high degree of fragmentation and low patch density have a greater effect on the quality of the habitats.

**Table 9:** Statistical description of the regression coefficients of the MGWR model.

Landscape index	Bandwidth	Mean	Min	Median	Max
PD	44	− 0.642	− 12.794	1.137	4.378
CONTAG	4670	1.062	1.052	0.003	1.066
AI	119	1.975	− 3.622	0.890	3.783
MESH	1894	− 2.296	− 2.950	0.478	− 1.538

The AI bandwidth was 119, or 19% of the sample as a whole. This led to a scale of action of approximately 7452 km^2^ and more significant geographic heterogeneity, suggesting that habitat quality sensitivity to landscape connectivity was still more relevant than patch density but less relevant than patch density. Good relationships between habitat quality and the majority of the agglomeration metrics were observed. The areas with negative correlations were primarily found in the western portion of Lan County's border connection, upstream Fangshan County, Louxian County, and the middle reaches of the southwest sections of Heshun County, Taigu County, and Qixian County. From the central plains to the surrounding regions, the spatial correlation gradually decreased.

Overall habitat quality has a significant negative correlation with the effective grid size, spatial heterogeneity is low, and the sensitivity of habitat quality to the effective grid size is lower than that of patch density and agglomeration index and has a greater influence. The bandwidth of the MESH is greater than that of the patch density and agglomeration index, which is 1894 and accounts for 31% of the total sample. Therefore, the scale of action is approximately 12,159 km^2^. The results indicate that, in the upper reaches with higher elevations, the habitat quality of the high-value area accounted for the largest proportion of the region, the intensity of human disturbance was low, the landscape connectivity was strong, and the impact of the negative correlation was weak. In contrast, the plain region in the middle reaches was dominated by arable land and construction land, the northeastern region in the middle reaches had uneven topography, and the negative correlation was weaker in the upstream region. Although the level of human activity was low, the negative association between the effective grid size and habitat quality grew steadily from west to east.

With a scale of action of approximately 23,142 km^2^ and little spatial variability, CONTAG had a maximum bandwidth of 4670 km^2^, accounting for 59% of the sample as a whole. This suggests that habitat quality is not very susceptible to the spreading index and has a wide range of effects. The findings typically indicated a slow increase in both the northeast and southeast directions from the center. Although it is evident that higher habitat quality is correlated with higher spreading indices, this relationship is not very important.

## Conclusions and discussion

### Conclusion

With several tributaries and a large basin, the Fen River traverses both northern and southern Shanxi Province and is crucial to the province's sustainable development. Significant changes have been made to the land use and landscape pattern, as well as the ecological quality of the Fen River Basin, as a result of the development of the natural environment, the acceleration of urbanization, and the implementation of numerous policies and regulations.

1. The overall habitat quality in the Fen River Basin was "low in the middle and high around the edges", with the largest decline occurring between 2005 and 2010 and a slowdown in the decline following that year. The spatial distribution of the habitat had more pronounced regional differences, and its spatial differentiation characteristics essentially corresponded with the current land-use situation.
2. The MGWR model fit findings were superior to those of the OLS model and the traditional GWR model, and the MGWR model was able to more clearly illustrate the various roles that habitat quality and landscape pattern indices play in one another. There were many driving mechanisms for habitat quality in PD, CONTAG, AI, and MESH. PD had the greatest impact on habitat quality, followed by AI, MESH, and CONTAG, which had the least impact. The hierarchy of influences was as follows: CONTAG had a positive single effect on habitat quality, MESH had a significant negative effect, and PD and AI had positive and negative bidirectional effects, respectively, on habitat quality.

### Discussion

The InVEST–MGWR coupled model was used in this study to assess the landscape pattern index of the Fen River Basin, to identify the spatial and temporal evolutionary features of habitat quality and to determine the effects of changing landscape patterns on habitat quality. In China and other nations, it offers a scientific resource for initiatives related to landscape design and biodiversity conservation.

1. According to results that were in line with those of other studies, habitat quality in the Fen River basin exhibited a declining trend year over year, with the worst decline occurring between 2005 and 2010 and a slower decline after that^20^. This is connected to the ecological environmental protection policies that Shanxi Province is implementing. When paired with the history of watershed governance, the scale of these efforts in 2015 was relatively limited. The "Fen River as the focal point of the 'seven rivers' ecological conservation and restoration of the overall program," "Fen River Basin Ecological Restoration Planning (2015–2030)" and other governance initiatives were announced in Shanxi Province in 2015. The initiatives were implemented in succession, strengthening the basin's overall land-use planning and ecological restoration. Although the ecological protection and restoration project of the mountains, forests, fields, lakes, and grasslands, which started in 2018, has not yet been able to fully explain the positive significance of improving ecological land use for the ecological environment, it has been able to fully explain the changes in habitat quality from the results of this study.
2. Overall, habitat quality was positively and negatively impacted "bidirectionally" by PD and AI, positively and negatively impacted single-handedly by CONTAG, and significantly negatively correlated with MESH. The increased density of low-habitat-quality patches means that they are more severely affected by the intensity of human activity, reducing the space available for high-quality areas. Additionally, habitat quality is more sensitive to patch density, and the negative effects of patch density on habitat quality are exacerbated as urbanization increases, while the negative effects of patch density on habitat quality are exacerbated due to building land. These are just a few of the ways that the increase in PD harms areas based on low habitat quality, such as urban centers. An increase in the extent of built-up land reduces the benefits. In most cases, the AI and habitat quality exhibited a positive correlation. The agglomeration index also had a major impact on enhancing habitat quality, with greater habitat quality observed in places with high ecological land assembly. In high-habitat areas, a lower MESH enhanced the resistance of high-quality patches to the effects of low-habitat patches, hence improving the quality of the habitat. The more habitat quality is positively impacted by the spread index, even though it is not as sensitive to this metric, the more CONTAG rises gradually from the center toward the northeast and southeast.

In summary, in metropolitan areas that rely on built-up land, decreasing the number of high-density built-up land patches and dispersing built-up land as widely as feasible are recommended. Promoting habitat quality involves embedding high-habitat patches, such as woodlands, and enhancing their connection while decreasing the density of building land patches, which lessens their detrimental effects on habitat quality. A higher agglomeration index can act as a buffer against habitat decline in peri-urban areas at the edge of urbanization, where construction land patches are continuously transforming the original arable land and the landscape pattern is more complex. The level of agglomeration in rural towns and cities should also be increased and maintained for areas of intermediate and high habitat, such as woodlands and water bodies, to prevent the fragmentation of landscapes from disrupting the continuity between habitats, which can lead to a decline in habitat quality. Simultaneously, lowering landscape complexity in low-habitat areas should decrease the probability of low-habitat patches. To prevent low-habitat patches from encroaching on and fragmenting high-habitat areas, it is also necessary to decrease the connectedness and agglomeration of these patches, such as construction land.

## Data Availability

The data that supports the findings of this study are available from the corresponding author upon reasonable request.
